# News Coverage of Older Drivers’ Fatal Car Crashes: Is It Over-represented?

**DOI:** 10.2188/jea.JE20240299

**Published:** 2025-05-05

**Authors:** Masao Ichikawa, Rie Tanaka, Akito Nakanishi, Yukie Sano

**Affiliations:** 1 Department of Global Public Health, Institute of Medicine, University of Tsukuba, Ibaraki, Japan; 2 Graduate School of Science and Technology, University of Tsukuba, Ibaraki, Japan; 3 Institute of Engineering, Information and Systems, University of Tsukuba, Ibaraki, Japan

**Keywords:** aging, drivers, newspaper article, traffic crashes

## Abstract

**Background:**

Japan’s stringent licensing policies for older drivers have not been questioned, possibly due to negative perceptions of older drivers potentially influenced by media coverage of their car crashes. We examined whether older drivers’ fatal crashes are over-represented in news articles.

**Methods:**

To examine the news coverage of fatal crashes that occurred between January 2016 and December 2020, we extracted driver- and crash-related data from articles reporting fatal crashes in the two best-selling newspapers, Yomiuri and Asahi. We obtained the corresponding data of police-reported fatal crashes during the same period. We calculated the proportion of newspaper-reported fatal crashes to police-reported fatal crashes by at-fault driver’s age group and crash characteristics.

**Results:**

Of 12,987 police-reported fatal crashes, 5,888 (45%) and 2,909 (22%) crashes were reported in Yomiuri and Asahi newspapers, respectively. Excluding 2,098 crashes where at-fault drivers or their ages were not identifiable, Yomiuri reported 39%, 35%, and 31%, and Asahi reported 20%, 16%, and 14% of fatal crashes caused by drivers aged <30 years, 30–69 years, and 70 years or older, respectively. Crashes that caused more fatalities or killed children tended to be reported regardless of at-fault drivers’ age groups. Compared with young and middle-aged drivers, older drivers’ fatal crashes involving child fatalities were more often reported, whereas their single fatal crashes ending in their own deaths were less often reported.

**Conclusion:**

Older drivers’ at-fault fatal crashes were not over-represented in the news coverage of overall fatal crashes, and their crashes killing themselves were under-reported.

## INTRODUCTION

In recent decades, licensing policies for older drivers have been strengthened in Japan.^1^ In 1998, mandatory driving lessons at license renewal were introduced for drivers aged 75 years or older, and this requirement was extended to those aged 70 years or older in 2002. Additionally, cognitive screening tests at license renewal became mandatory for drivers aged 75 years or older in 2009. From 2017, these tests are required anytime they violate certain regulations, such as wrong-way driving and ignoring traffic signals. Moreover, those suspected of having dementia during screening are required to see physicians, and a confirmed dementia diagnosis leads to license revocation. From 2022, drivers aged 75 years or older who have violated certain regulations in the past 3 years must take a road test at license renewal, in addition to driving lessons and cognitive screening tests.

Stringent licensing policies for older drivers have not been questioned, possibly due to negative perceptions of older drivers and misunderstandings of their crash risk. An internet panel survey conducted by the National Police Agency in 2019 revealed that 80% of 2000 age- and sex-balanced respondents believed that licensing for older drivers should be further revised.^2^ The National Police Agency explained that the policies for older drivers have been strengthened in consideration of their physical and cognitive declines and in response to their high-profile crashes.^1^ However, despite the increasing number of older drivers, the number and the rate (per driver) of their at-fault crashes are still lower than those of young drivers.^3^ Moreover, the risk of injuries imposed on collided counterparts is no greater in older drivers’ at-fault crashes than in crashes caused by drivers in other age groups.^3^

Public perceptions of older drivers are potentially influenced by media coverage and framing of their crashes according to framing effects theory^4^ and theory-based experiments.^5^ In Japan, a content analysis of opinion articles regarding older drivers in four major Japanese newspapers, published from 2006 to 2019, found an abrupt increase in opinion articles on older driver-related countermeasures, especially in response to high-profile crashes caused by older drivers, particularly those involving child fatalities.^6^ Our previous study, analyzing the contents of approximately 2.2 million tweets regarding older drivers in Twitter (currently X) from 2010 to 2021, found that negative tweets had increased despite the decreasing trend of older drivers’ crashes in recent years.^7^ This raised the question of whether older drivers’ crashes are over-represented in news articles. In the present study, we examined this possibility, considering factors such as the number of fatalities in the crash, child fatalities in the crash, collided counterparts, multiple-vehicle crashes, and traffic violations.

## METHODS

### Data

To examine the news coverage of fatal car crashes that occurred between January 2016 and December 2020, we extracted data from articles in the two best-selling newspapers, Yomiuri Shimbun and Asahi Shimbun, published between January 2016 and January 2021.

First, two research assistants independently searched for relevant articles in the respective newspaper database using three broad terms: “jiko” (accident), “shibou” (death), and “kuruma” (car) to cover all potential articles. They then reviewed the identified articles to exclude those not reporting fatal crashes (such as opinion articles) and extracted data from the remaining articles on the date of crashes; collided counterparts (pedestrians, bicycles, motorcycles, cars, trains, objects, others such as carts, and none); at-fault drivers’ age, sex, and traffic violations (hit-and-run, drunk driving, distracted driving using mobile phones and navigation systems, and dangerous tailgating); whether multiple vehicles were involved; whether pre-school, elementary, or junior high school children were killed; and whether at-fault drivers, their passengers, or collided counterparts were killed in each crash. Follow-up articles on fatal crashes were used to extract additional data, but the same crashes were counted as one crash. Subsequently, the data extracted by the two research assistants were merged, and discrepancies between the datasets were resolved by the first and second authors. When at-fault drivers were not identifiable in the articles, we excluded these crashes from the main analysis but included them in a sensitivity analysis, assigning drivers of the car without fatalities as at-fault drivers (ie, the collided counterparts had fatalities).

We obtained the corresponding data of fatal crashes that occurred during the same period from the Institute for Traffic Accident Research and Data Analysis, which compiles traffic crash data of the National Police Agency.

### Analysis

To determine whether fatal crashes caused by older drivers were over-represented in the news coverage compared with those caused by young and middle-aged drivers, we calculated the proportion of newspaper-reported fatal crashes to police-reported fatal crashes by at-fault driver’s age group (<30 years, 30–69 years, and 70 years or older). We used this age group because at-fault crash rates for drivers aged <30 years are much higher than the rates for drivers in other age groups, whereas the rates for drivers aged 30–69 years are stable, the rates start to increase around the age group of 70 years or older,^3^ and drivers aged 70 years or older are subject to the licensing policies explained earlier. We also calculated this proportion by the number of fatalities in the crash, child fatalities in the crash, collided counterparts, multiple vehicle crashes, and traffic violations because potential over-representation might depend on these factors. If a driver committed multiple violations (eg, hit-and-run and drunk driving) in a crash, we counted it as one for each violation. The calculations were conducted separately for Yomiuri Shimbun and Asahi Shimbun newspapers to identify any differences in coverage between the two newspapers. Finally, we conducted the aforementioned sensitivity analysis.

## RESULTS

Between January 1, 2016, and December 31, 2020, there were 12,987 police-reported fatal crashes, of which 5,888 (45%) and 2,909 (22%) were reported in Yomiuri Shimbun and Asahi Shimbun newspapers, respectively. However, at-fault drivers were not identifiable in 1,033 and 539 fatal crashes reported in the respective newspapers. In the remaining 4,855 and 2,370 fatal crashes, the at-fault driver’s age was not identifiable in 301 and 225 fatal crashes, respectively. Excluding these crashes (2,098 out of 8,797), Table 1 shows the coverage by at-fault driver’s age group and crash characteristics. Yomiuri reported 39%, 35%, and 31%, and Asahi reported 20%, 16%, and 14% of fatal crashes caused by drivers aged <30 years, 30–69 years, and 70 years or older, respectively.

**Table 1.** Number of police-reported and newspaper-reported fatal crashes, and the proportion of fatal crashes covered by two major newspapers, by at-fault driver’s age group and crash characteristics, from 2016 to 2020

	Police-reported			Newspaper-reported (Yomiuri Shimbun)							Newspaper-reported (Asahi Shimbun)						
		
	At-fault drivers’ age			At-fault drivers’ age							At-fault drivers’ age						
		
	<30 years	30–69 years	≥70 years	<30 years		30–69 years		≥70 years		Unknown	<30 years		30–69 years		≥70 years		Unknown
										
	*n*	*n*	*n*	*n*	%	*n*	%	*n*	%	*n*	*n*	%	*n*	%	*n*	%	*n*
All crashes	2,058	8,092	2,837	805	39%	2,860	35%	889	31%	301	420	20%	1,327	16%	398	14%	225

Number of fatalities in the crash																	
1	1,984	7,941	2,742	750	38%	2,763	35%	843	31%	285	380	19%	1,252	16%	373	14%	204
2	59	134	91	43	73%	83	62%	43	47%	11	29	49%	61	46%	22	24%	19
3 or more	15	17	4	12	80%	14	82%	3	75%	5	11	73%	14	82%	3	75%	2

Children killed in the crash																	
Pre-school children	30	71	10	13	43%	34	48%	5	50%	4	13	43%	25	35%	4	40%	2
Either pre-school or elementary school children	36	153	22	17	47%	68	44%	13	59%	9	15	42%	58	38%	11	50%	4
Either pre-school, elementary or junior high school children	46	169	23	17	37%	73	43%	14	61%	14	17	37%	62	37%	13	57%	4

Collided counterparts																	
Pedestrians	786	3,666	689	281	36%	1,223	33%	223	32%	146	117	15%	510	14%	81	12%	103
Bicyclists	176	855	160	73	41%	306	36%	66	41%	46	25	14%	118	14%	21	13%	18
Motorcyclists	216	743	128	40	19%	103	14%	12	9%	11	18	8%	46	6%	7	5%	6
Cars	465	1,409	779	177	38%	582	41%	223	29%	49	127	27%	350	25%	118	15%	54
Trains	2	19	25	2	100%	15	79%	25	100%	1	2	100%	13	68%	17	68%	5
Objects	363	1,065	648	163	45%	424	40%	221	34%	24	91	25%	189	18%	106	16%	14
Others^a^	4	15	1	0	0%	0	0%	0	0%	0	0	0%	0	0%	0	0%	0
None	46	320	407	69	150%	207	65%	119	29%	24	40	87%	101	32%	48	12%	25

Multiple vehicle crashes	283	1,048	360	46	16%	170	16%	50	14%	20	29	10%	123	12%	35	10%	24

Traffic violations																	
Hit-and-run	163	432	73	48	29%	139	32%	27	37%	16	30	18%	91	21%	13	18%	20
Drunk driving	233	585	72	38	16%	72	12%	0	0%	1	26	11%	37	6%	1	1%	2
Using mobile phones	52	115	4	4	8%	14	12%	0	0%	0	3	6%	15	13%	0	0%	0
Using navigation systems	36	64	3	1	3%	2	3%	0	0%	0	1	3%	3	5%	0	0%	0
Dangerous tailgating	2	7	0	2	100%	3	43%	0	n/a	0	2	100%	3	43%	0	n/a	0

n/a: not applicable because the denominator is 0.

^a^Others include carts and unknown counterparts.

In both newspapers, the higher the number of fatalities in the crash, the more likely such crashes were reported, regardless of at-fault drivers’ age. However, the coverage of crashes involving older drivers and killing two people was less than that of other drivers, which was not the case for crashes killing one or three or more people. Crashes involving child fatalities were more likely to be reported than those without child fatalities, and the coverage of such crashes was greater for older drivers compared to other drivers.

Regarding collided counterparts, crashes involving trains were highly covered, though the number of such crashes was small. Crashes involving pedestrians and bicycles, as well as multiple vehicle crashes, were reported to a similar extent across all at-fault drivers’ age groups. However, the coverage of crashes involving cars, motorcycles, and objects caused by older drivers was less than that of other drivers. Notably, crashes with no collided counterparts (ie, single fatal crashes) caused by older drivers were far less likely to be reported than those caused by other drivers, even though 14% (407/2,837) of all fatal crashes caused by older drivers were single fatal crashes, compared to only 2% (46/2,058) and 4% (320/8,092) among drivers aged <30 years and aged 30–69 years, respectively. The number of single fatal crashes among drivers aged <30 years reported by Yomiuri exceeded the police-reported number.

In terms of traffic violations, crashes involving hit-and-run were reported to a similar extent across at-fault drivers’ age groups, though crashes involving drunk driving among older drivers were less likely to be reported than those among other drivers. Crashes while using mobile phones and navigation systems and dangerous tailgating were rare among older drivers. The sensitivity analysis revealed similar results (not shown in the table).

## DISCUSSION

Older drivers’ at-fault fatal crashes were not over-represented in the news coverage of overall fatal crashes relative to young and middle-aged drivers in two major Japanese newspapers. However, differences in coverage across at-fault drivers’ age groups were observed when stratified by crash characteristics. Crashes causing more fatalities and involving child fatalities tended to be reported, regardless of the at-fault drivers’ age groups.

A unique tendency in the coverage of older drivers’ fatal crashes was the under-reporting of their single fatal crashes and crashes against objects, while coverage of their fatal crashes involving child fatalities was higher than that of other drivers. In single fatal crashes and crashes against objects, drivers or their passengers died, and such crashes occurred much more frequently among older drivers than other drivers. We previously reported that the proportion of at-fault driver deaths to all deaths in fatal crashes caused by older drivers is much higher than for other drivers.^3^ Moreover, the proportion of vulnerable road user deaths (ie, pedestrians and bicyclists) to all deaths in fatal crashes caused by older drivers is smaller than for other drivers.^3^ Nevertheless, fatal crashes killing others were more likely to be reported than those killing themselves among older drivers compared with other drivers, which might prompt people to perceive that older drivers harm others in their fatal crashes, despite many of their fatal crashes ending in their deaths.

We found it difficult to interpret the lower coverage of older drivers’ fatal crashes involving drunk driving and multiple fatalities, and the exceeding coverage of young drivers’ single fatal crashes. Generally, traffic violations were not widely reported in the news articles. In fact, the proportion of fatal crashes involving drunk driving among drivers aged <70 years reported in the news articles was as low as 6–16%, though this proportion was even lower among drivers aged 70 years or older. Additionally, the inaccurate coverage of young and older drivers’ fatal crashes mentioned above might be partly attributable to the definition of traffic crash deaths in the police data, where deaths are defined as those occurring within 24 hours of crashes. However, the National Police Agency collects information on traffic crash deaths occurring within 30 days of crashes for international comparison, and 17% of traffic crash deaths occurring within 30 days of crashes happened after 24 hours of crashes during the 5-year study period.^8^ In the news articles, it is usually not reported whether deaths occurred within 24 hours of crashes, and we likely included deaths that occurred after 24 hours of crashes. This might have resulted in the inaccurate coverage of fatal crashes by the crash characteristics.

We acknowledge the study’s limitations. First, misidentification of at-fault drivers and crash characteristics could occur during data extraction if two research assistants happened to extract the data incorrectly from the same news articles. The extent to which this occurred is uncertain. However, if it occurred, the results in the two newspapers could be inconsistent, but they appeared to be similar. Second, we could not identify driver’s age among 301 of 4,855 (6%) and 225 of 2,370 (9%) at-fault drivers in Yomiuri Shimbun and Asahi Shimbun newspapers, respectively. However, this is not a problem because we aimed to examine the extent to which fatal crashes were covered by newspapers across at-fault driver’s age groups. Moreover, this would not discriminatively influence readers’ perception of older drivers because the drivers’ ages are unknown. Third, we did not examine local newspapers that may have reported fatal crashes that were not covered by national newspapers. Additionally, we did not investigate other forms of mass media, such as television, magazines, and news websites. While national newspapers are expected to cover high-profile fatal crashes, our findings would be distorted if the characteristics of crashes reported in national versus local newspapers or other mass media differed largely.

This study focused solely on the representation of older drivers’ crashes in news coverage. Future research should explore how the coverage and framing of older drivers’ crashes across various mass media platforms, including social media, influence public perceptions of older drivers and licensing policies. Previous studies indicated that mass media plays a crucial role in road safety by highlighting these issues to the public and policymakers, prompting actions to improve road safety.^9^^–^^11^ Such research would help shaping unbiased public perceptions of older drivers, leading to sound licensing policies. It is important to consider that licensing policies may impact the life course of older drivers, as driving cessation can cause adverse social and health outcomes.^12^^–^^14^

In conclusion, older drivers’ at-fault fatal crashes were not over-represented in the news coverage of overall fatal crashes. However, their fatal crashes involving child fatalities were more often reported, whereas their single fatal crashes were less often reported than those of young and middle-aged drivers.
